# Albuminuria

**Published:** 1893-02

**Authors:** W. L. Reed

**Affiliations:** St. Louis, Mo.


### BUREAU OF CLINICAL MEDICINE.

CAROLINE E. HASTINGS, M. D., CHAIRMAN.

### ALBUMINURIA.

W. L. REED, M. D., ST. LOUIS, MO.

At our annual meeting at Watch Hill, I reported a serious case of albuminuria, which was effectually cured with Natrum-muriaticum. The young man is ruddy, and has learned black-smithing.

I now present a case which has some striking similarities to the above-mentioned case in the character or manner of the swelling or dropsical effusion.

On the 24th day of November, 1890, was summoned to Edwardsville, Ill., in consultation with Dr. W. Enos, a true homœopath, to see his brother-in-law, who had been ailing some four months with an anasarcous swelling. The whole body was swollen, but some places much worse than others. The lower extremities were so large that large sized pants had to be ripped and held together with tapes. Arms and fingers swollen; fingers spread apart, and he could not shut his hand. The body and face seemed to be swollen in rolls, or patches. There was no thirst. Had numbness and heat of ears on lying on them. Cold feet; cold legs to knees. Swelling in extremities was worse at night than during the day. Itching all over the body, without any eruption, worse on undressing. He had scanty urine, of a light dirty color, specific gravity 1010. Nitric-acid and heat, and Picric-acid revealed immense deposits of albumen. The test-tube, after standing several days, would be half full of amount of fluid. The microscope showed hyaline and blood casts in great quantities. This case was remarkable in the paucity of symptoms. I gave him four powders of Natrum-mur.$^{200}$, one to be taken every evening. The indications for Natrum were so very meagre, I based it upon the fact that he has been sleeping in open depots in Wyoming, being exposed to malarial influences. One week's treatment showed great improvement in the diminution of the amount of albumen, and a like subsidence of the swelling. Sac-lac. for one week. Repeat the Natrum, M potency, B. & T. He was continued under the influence of this till the 22d day of December. The case making no improvement upon the first week's treatment, after a month's treatment, he received a dose of Hepar-sul. Was continued on this till January 17th. He then received one dose of Natrum-mur., M potency, with a good and satisfactory diminution of albumen and swelling. Was on this remedy without repetition till February 14th. We were then informed by the patient that whilst employed in a civil engineer corps, in Wyoming, he had a rash on his body ; had been exposed to a case of scarlet fever. From the exposure he was subject to, the rash suddenly disappeared. In a short time, noticed his body began to swell. I, at once, gave him one dose of Bryonia$^{70m}$, Fk. In a few days, the repurcussed rash was reproduced. It was on his body for one day, during which time he took a bath, when the rash suddenly disappeared. Bryonia$^{cm}$, H. S., one dose, but was never able to reproduce the rash again. Was under Bry. till the 17th day of March. There was great variableness as to the amount of albumen each day. He now detailed another peculiar condition. Whenever he would become intently interested in a game of chess, or allow the little children to romp on his knees, there would be a decided increase in the amount of albumen. I now gave him a dose of Arnica$^{cm}$, H. S., with most remarkably good results. He was under the influence of this remedy till the 30th day of May. The swelling had entirely disappeared under Arnica. But the amount of albumen showed a varying condition, increasing and diminishing daily from one to three grammes. I now gave one dose of Natrum-mur.$^{cm}$, Fk. ; was under the influence till August 1st. For some condition in his organism that I had forgotten to make a note of, I gave him a dose of Phos.$^{cm}$, Fk. There was now daily from one and one-half to two and one-half grammes of albumen to the litre, according to Esbach's albuminometer. This proved to be my last prescription for this case. A short time after, he called and stated to me that he desired to consult Dr. H. C. Allen, of Chicago. I was only too willing, and gave the doctor an extensive *résumé* of the case, as to how I had treated it. Dr. Allen writes that he placed the patient, August 16th, on the complement of Natrum-mur., Apis$^{cm}$, one dose. September 1st, one dose of Apis$^{40m}$; slight improvement with each dose. September 10th, improvement ceased: Sulphur$^{cm}$, one dose. October 9th, no results; no symptoms; no change at all. Natrum-m.$^{1m}$, some gain. No effect on amount of albumen. December 10th, Phos.$^{cm}$, under which general health improved, but little change in albumen. Decided now to go to work, and stop all treatment. I saw him some time since resuming work; says he still secretes from one to one and one-half grammes of albumen in twenty-four hours. He is busily engaged every day. Has a good appetite, sleeps well, and says he feels as well as he ever did. This condition in his organism may continue for years. The tubuli uriniferi have been denuded of epithelium. This epithelium must be restored before the kidneys can perform their proper functions. There is no psoric taint in his organism. There can be little doubt but he will be completely restored to health.

### Discussion.

Dr. E. W. Sawyer—In the Mississippi Valley, I find a large proportion of the cases of albuminuria come from the abuse of Quinine in malarial fevers. It is astonishing how small a dose of Quinine will suppress these cases of intermittent fever. This explains to me why Nat-mur. cures so many of these cases, it being the antidote to Quinine. It is my opinion that if Dr. Reed had given that case a dose of Quinine high, there would not have been that lack of symptoms of which he complains.

Dr. Reed—The young man never took Quinine in his life; was never under the treatment of an old-school physician; and hence, what Dr. Sawyer says falls to the ground.

Dr. Hoyne—I expected Dr. Reed was going to cure that case with one dose, from what he said yesterday. Instead I find he gave four doses before stopping. In treating a case like that I give a remedy and wait from one-half to twelve hours, and repeat or change as required, and Dr. Reed criticised me for it. And now to-day Dr. Reed gives four doses in repetition without waiting for change of symptoms or anything else.

Dr. Reed—I gave the four doses because it was a comparatively low potency. When I give a CM potency they only get one dose. Sometimes when I use a low potency I do repeat.

Dr. J. H. Allen—My experience in these cases is that Quinine is an important factor. I had a case very similar to Dr. Reed's. The urine had the tube-casts and albumen in abundance. Old-school physicians had treated him first and he got Quinine by the ounce. It broke up his chills. About two years after he got this kidney trouble. My method was to look up the case of malaria and find what was his remedy at that time. I gave him a dose of Chin-sulph. high, and it cured him. The same physicians examined his urine who had found the tube-casts and albumen and pronounced it perfectly normal.

